# Dr. A. J. Volck's Seventieth Birthday. A Characteristic Event

**Published:** 1898-06

**Authors:** 


### Editorial, Etc.

### DR. A. J. VOLCK'S SEVENTIETH BIRTHDAY. A CHARACTERISTIC EVENT.

There is published elsewhere in the Journal the proceedings at a complimentary dinner by the Association of Dental Surgeons of Baltimore to Dr. Adalbert J. Volck in commemoration of his seventieth birthday, April 14, 1898. While not prompted by the reason given for the complimentary dinner to Dr. Geo. H. Cushing, April 27th, in Chicago, who retires to rural pursuits to regain his health after a long and honorable career of forty-one years in that city, yet the spirit that animated the dentists in both cities is the same—a recognition of personal friendship and an evidence of good will.

The Association of Dental Surgeons was incorporated October 16, 1888, and was managed the first year by its founders, Richard Grady, Wm. A. Mills, Wm. S. Twilley, Charles E. Duck and Adalbert J. Volck. It has held monthly meetings regularly since that date at which papers have been read and incidents of office practice discussed. Annual summer fishing trips have also been participated in by members.

There is a superstition that dentists are not a particularly united body, that they are not invariably and inseparably attached to one another. A grain or so of truth must be conceded to that superstition; but in the Association of Dental Surgeons, which was founded in the belief that dentists could hold business and social meetings in which good fellowship should prevail, "there is not," to quote the words of Dr. Volck, "a blot or blemish on the brotherly unison of the members;" and this sentiment is echoed with pride and thankfulness by the members, who talk among themselves without first calculating that what they say will be repeated or misrepresented to their disadvantage. Sneakiness and underhand dealing are unknown arts in the meetings. The members make allowances for the pettiness of human nature and treat one another with great cordiality. There is no bickering of any kind. Prejudice has nowhere an abiding place.

There is something rather awful implied in certain words of Dr. Volck—"sympathy for a man who has borne the burdens of professional labor for so many years [upwards of forty-five years in Baltimore alone] without ever having disgraced his profession." They make us think of ourselves and of our friends, and of how it is with us who are going on (in most cases) very much as we probably shall go as long as we live. There is no saying how the page of our life may be blotted before it is finished. Very many men live on, just to disappoint. They have done their best already, and they are now producing work inferior to what they once did, to what we might expect of them. There are other men who go on through life without deterioration, men who are always up to the mark; and this may be said of Dr. Volck respecting his artistic gifts as shown in the Appold and Hooper testimonials, after his reputation as a dentist had made his name familiar.

Dr. Volck is one of the few men who in their own lives see their names celebrated. He has taken a foremost place and is known as the Nestor of the dental profession in Baltimore. Dr. Volck's clear, broad views and well-stored mind make him an attractive and interesting ccmpanion. The whole manner of the man impresses one with his open, frank good-nature, and his conversation reveals a mind thoroughly trained. He possesses to a singular degree the power of presenting subjects clearly and forcibly, and almost unconsciously uses that "universal language of the world," drawing, when troubled to make himself understood.

It is but fair to say, in conclusion, that Rev. Johannes A. Oertel wrote "Early Reminiscences of Dr. Volck;" Gen James Howard wrote "Dr. Volck as a Man;" Mr. Thomas Hedian wrote "Dr. Volck as an Artist," and Col. Wm M. Pegram wrote the poem, "To Dr. A. J. Volck on his Seventieth Birthday," which concludes with the toast, adopted as the voice of the Association :

"Through joys or sorrows, aye through smiles or tears,
God give thee strength unto thy Fourscore years."

